# Telomere Length at Recruitment Predicts Lifespan and Lifetime Reproductive Success in the Barn Swallow ( *Hirundo rustica* )

**DOI:** 10.1111/mec.70496

**Published:** 2026-08-05

**Authors:** Andrea Novelli, Manuela Caprioli, Roberto Ambrosini, Alessandra Costanzo, Diego Rubolini, Marco Parolini, Andrea Romano

**Affiliations:** ^1^ Department of Environmental Science and Policy University of Milan Milan Italy

**Keywords:** barn swallow, lifespan, lifetime reproductive success, sexual ornaments, telomere attrition, telomere length

## Abstract

Identifying molecular mechanisms linked to variation in lifespan, reproductive output and sexually selected traits is key to understanding life‐history evolution in wild populations. Telomere length (TL) and attrition (ΔTL) have been proposed as biomarkers of somatic state and maintenance (survival). Yet evidence linking telomeres to lifetime fitness and ornament expression remains limited. We quantified age‐related variation in TL and annual ΔTL between the year of recruitment (1‐year‐old) and death, as well as tested whether TL at recruitment predicts realized lifespan and lifetime reproductive success in a short‐lived migratory passerine, the barn swallow ( *Hirundo rustica* ). We also examined associations between TL/ΔTL and two sexual ornaments (outermost tail feathers length and ventral plumage colouration). TL constantly declined with age, and longer TL as yearling was linked to increased lifespan and higher number of fledglings during the entire life, indicating that TL captures considerable variation in individual quality. Moreover, longer tail feathers and a larger increase in tail length between consecutive years were associated, respectively, with shorter TL and faster ΔTL among females. Additionally, plumage darkening between consecutive years was associated with reduced ΔTL in both sexes, indicating that telomeres covary with secondary sexual traits in a sex‐ and trait‐specific manner and may be involved in sexual selection processes. Overall, even in a species with high annual mortality, TL positively predicted major fitness components, supporting TL as a biomarker of individual quality and suggesting that longer telomeres at the onset of adulthood may be favoured by natural selection in wild populations.

## 1 Introduction

Explaining the mechanisms shaping inter‐individual differences in Darwinian fitness is a central goal in evolutionary ecology. Although fitness components are expressed at the organismal level as the combination of survival and reproductive success, understanding why they differ among individuals requires considering the physiological processes linked to individual somatic quality and, ultimately, performance. Identifying molecular mechanisms that capture this physiological variation is therefore crucial for understanding how natural and/or sexual selection may have driven the evolution of species‐specific life‐history traits. In this context, telomeres, the nucleoprotein complexes at the ends of eukaryotic chromosomes known to preserve genome stability and integrity (Blackburn 1991, 2005), may play a pivotal role (Monaghan 2010, 2024; Monaghan et al. 2022).

In somatic cells, telomere length progressively decreases with each cell division due to the ‘end‐replication problem’, which occurs because DNA polymerases cannot fully replicate linear chromosomes (Harley et al. 1990; Blackburn 2005). In addition to the replicative history, the rate at which telomeres shorten also depends on the exposure to various environmental stressors (e.g., harsh climatic conditions, competition, parasite burden—Monaghan 2014; Costanzo, Parolini, et al. 2017; Eastwood et al. 2022; Badás et al. 2023; Eastwood, Dupoué, Verhulst, et al. 2023), as well as on metabolic rates (e.g., Monaghan and Ozanne 2018; Criscuolo et al. 2021). Both can increase cell division rates and/or reactive oxygen species production, thereby promoting oxidative stress to which telomeres can be particularly vulnerable (Haussmann and Marchetto 2010; Chatelain et al. 2020; Armstrong and Boonekamp 2023). Although the activity of the enzyme telomerase partially counteracts telomere attrition by adding new telomeric repeats (Greider and Blackburn 1989; Lingner et al. 1997), the above‐mentioned factors generally lead to telomere shortening with age, as shown across multiple vertebrate species using both cross‐sectional and longitudinal approaches (Remot et al. 2022). However, evidence of telomere maintenance or elongation under favourable physiological or environmental conditions suggests that telomere restoration may occur. While such cases are rare and context‐dependent in endotherms (Mizutani et al. 2013; Noguera et al. 2015; Brown et al. 2022), they are reported to be more common in ectothermic vertebrates, possibly reflecting the broader retention of telomerase activity across somatic tissues compared to endothermic vertebrates (e.g., McLennan et al. 2018; Voituron et al. 2023; Olsson et al. 2025).

Critically shortened telomeres trigger cell senescence or apoptosis, thereby impairing tissue renewal capacity and potentially leading to a progressive deterioration of individual phenotype and performance (Campisi et al. 2001; Deng et al. 2008). This suggests that telomere length and its attrition rate can serve as biomarkers of individual somatic state and investment in self‐maintenance. Indeed, they are increasingly investigated as predictors of survival in many taxa, with longer telomeres and/or slower telomere attrition associated with reduced mortality risk (Salomons et al. 2009; Boonekamp et al. 2014; Wilbourn et al. 2018). Moreover, this pattern is more likely to emerge when telomere length is investigated throughout an individual's lifespan because repeated survival outcomes across years may better reflect persistent differences in somatic state among individuals (Eastwood et al. 2019; Bichet et al. 2020; Sheldon et al. 2022; Vedder et al. 2022; Eastwood, Dupoué, Delhey, et al. 2023). However, the relationship between telomere length and lifespan is not consistently observed across studies in wild populations (Caprioli et al. 2013; Sudyka et al. 2019; Kauzálová et al. 2022; Pepke et al. 2022; Chik et al. 2024), likely because stochastic extrinsic mortality can weaken the link between physiological condition and survival (Monaghan 2010, 2024; Stier et al. 2014; Criscuolo et al. 2021; Pepke et al. 2023).

In iteroparous species, increased longevity may provide a higher number of reproductive opportunities; thus, lifespan often translates into higher lifetime reproductive success (Newton 1989). Therefore, if longer telomeres are associated with higher somatic quality, and this translates into increased longevity, they may also predict higher lifetime reproductive success, and thus be favoured by natural selection (Monaghan 2010, 2024; Monaghan et al. 2022). Accordingly, long‐term studies reported positive associations between telomere length and lifetime reproductive success (Pauliny et al. 2006; Eastwood et al. 2019; Heidinger et al. 2021; Chik et al. 2024). These studies are therefore particularly informative for understanding whether, and under which conditions, telomeres are relevant to life‐history evolution. However, they remain scarce mainly due to the logistical challenges of monitoring individuals throughout their lifespan in the wild (e.g., Pauliny et al. 2006; Eastwood et al. 2019; Sudyka et al. 2019; Heidinger et al. 2021; Morland et al. 2023; Chik et al. 2024). Furthermore, findings remain mixed across species because pace‐of‐life differences and ecological conditions can modulate the association between telomere length and lifespan (e.g., Tricola et al. 2018; Criscuolo et al. 2021), while also influencing the metabolic costs of reproduction (Bauch et al. 2016; Merkling et al. 2017; Noguera 2017; Costantini 2024), which are more likely to be captured by telomere dynamics rather than by telomere length measured at a single time point (Sudyka et al. 2019; Sudyka 2019).

The production and maintenance of secondary sexual traits to attract potential mates can represent a substantial component of reproductive costs (e.g., Huhta et al. 2003; Webster et al. 2018). However, sexual selection theory also suggests that secondary sexual traits function as honest signals of individual genetic/phenotypic quality and are used by the sex investing more in reproduction to select high‐quality mates (Andersson 1994; Nolazco et al. 2022). Under such a scenario, if telomere length and/or dynamics are a major component of variation in individual quality, they are expected to covary with the expression of secondary sexual traits. Alternatively, if telomere length and/or dynamics reflect the physiological cost of ornament expression, more elaborate traits would be associated with shorter telomeres and/or faster telomere attrition. Given these contrasting predictions, investigating the association between telomeres and secondary sexual traits becomes crucial in understanding the molecular mechanisms underlying sexual selection processes. However, previous studies are limited and have provided conflicting evidence, from positive (Parolini et al. 2017; Taff and Freeman‐Gallant 2017) to negative (Azcárate‐García et al. 2020; Kauzálová et al. 2022) or no association (Morosinotto et al. 2022; Taylor et al. 2024) between telomere and sexually selected traits.

We conducted a longitudinal study of telomere length and dynamics in a short‐lived long‐distance migratory passerine bird, the barn swallow ( *Hirundo rustica* ). The aim of the study was to evaluate their relationships with lifespan, lifetime reproductive success and the expression of secondary sexual traits, and thus assess whether and how telomeres are associated with fitness in a short‐lived species where stochastic mortality is expected to be high. Specifically, we focused on breeding adults (≥ 1‐year‐old) monitored throughout their entire lives, and we aimed to: (1) examine variation in telomere length and its annual change according to age; (2) assess whether telomere length at recruitment (yearlings = 1‐year‐old individuals) predicts lifespan and lifetime reproductive success; and (3) investigate the association between telomere length/annual change in telomere length and the expression of secondary sexual traits, specifically the length of the outermost tail feathers (hereafter, tail length) and ventral plumage colouration, that are well‐established sexually selected traits in the barn swallow (Costanzo, Ambrosini, et al. 2017; Romano, Saino, and Møller 2017; Romano, Costanzo, et al. 2017).

## 2 Material and Methods

### 2.1 Study Organism

The barn swallow is a short‐lived, long‐distance migratory and socially monogamous passerine bird (Turner 2006). Italian populations breed semi‐colonially in rural buildings from April to August and spend the non‐breeding season south of the Sahara (Turner 2006). Breeding adults (≥ 1‐year‐old) show strong breeding philopatry, returning to the same colony in consecutive years (Turner 2006; Romano et al. 2025). Females lay 1–3 clutches per season with 1–7 eggs each (modal size: 5 eggs). They lay one egg per day and incubate for approximately 14 days. After hatching, both parents provide parental care to the offspring, which fledge after ca. 20 days (Turner 2006). Adults show moderate sexual dimorphism, with males typically displaying darker ventral plumage colouration and longer outermost tail feathers than females (Turner 2006; Saino, Romano, et al. 2013). Both traits are under sexual selection, with longer tail feathers and darker ventral plumage associated with higher viability and fitness (Costanzo, Ambrosini, et al. 2017; Romano, Saino, and Møller 2017; Romano, Costanzo, et al. 2017). Additionally, these traits show age‐dependent variation in both sexes, with individuals becoming darker and developing longer tail feathers as they age (Møller 1991; Romano, Costanzo, et al. 2017). Independent of these age‐related patterns, ventral plumage colouration and outermost tail feathers length can also vary between years within the same individual, as they are both renewed annually during the complete plumage moult performed in the African non‐breeding grounds (Møller et al. 1995; Saino, Romano, Caprioli, et al. 2013).

### 2.2 Field Procedures

We studied three cohorts (2012 to 2014) of breeding barn swallows from recruitment as adult breeders (i.e., yearlings: age = 1‐year‐old) at five colonies (= farms) located south‐east of Milan (average coordinates: 45.30° N, 9.50° E), in northern Italy, until the death of the last individual included in the study. In all study years (2012–2020), between April and June, we performed at least two capture sessions of breeding adults, during which they were marked with univocally numbered metal and plastic colour rings (details in Saino et al. 2012; Romano et al. 2025). As we exhaustively captured almost all adults within colonies (proportion of unmarked individuals at the end of the breeding seasons over 30 years of captures: ~5%; Romano et al. 2025) and barn swallows of our population exhibit strong breeding site fidelity, as shown by the virtually nihil breeding dispersal (0.02%; Romano et al. 2025), newly captured individuals in any year_*i*_ were considered yearlings that were born the year_*i*−1_ in other colonies, except for the rare cases of local recruits (individuals ringed as nestlings and returned to their natal colony to breed: ~5% mostly males; Novelli et al. 2026). Thus, age could reliably be assigned based on the year of first capture (details in Saino et al. 2012; Romano et al. 2025). Along the same way of reasoning, individuals captured in year_*i*_, but not recaptured in year_*i*+1_, were assumed to be dead (details in Saino et al. 2012; Romano et al. 2025). Lifespan was therefore accurately estimated as the number of years elapsed between the year preceding the first capture (i.e., the hatching year) and the last year the individual was observed in the colony (e.g., an individual first captured in 2013—and therefore hatched in 2012—and last recaptured in 2015 was assigned a lifespan of 3 years).

Upon capture, standard measurements were performed on different morphometric traits (i.e., tail length), and adults were sexed according to morphological and behavioural traits (incubation behaviour, performed by females only in the study population; Turner 2006). We also collected contour feathers from the ventral region and blood samples from almost all individuals in each year of life for later spectrometric colour analysis and telomere length assessment, respectively (Saino, Canova, Costanzo, et al. 2013). Blood was collected from the brachial vein using heparinized capillary tubes that were immediately placed in cool bags in the field. Blood samples were then transported to the lab for centrifugation at 11,500 rpm for 15 min to separate red blood cells from plasma and were finally stored at −20°C within 3–4 h after collection.

In all study years, except for 2018, along with capture sessions, we regularly (at least twice a week) inspected all the nests to record breeding events within the season. In addition, we assigned most adult breeders to their clutches by observing their univocal colour ring markings. We were thus able to quantify the seasonal reproductive success of most individuals, expressed as the sum of the number of nestlings fledged in each brood of the individual within a breeding season. For a large number of individuals, we were able to collect seasonal reproductive success across all breeding seasons of their lives, thereby allowing us to estimate lifetime reproductive success (i.e., number of fledglings produced during the entire lifespan; hereafter, LRS). However, since extra‐pair fertilizations are known to occur in this species (Turner 2006), including in the study population (12.7%; Costanzo, Ambrosini, et al. 2017), the estimated LRS for males corresponds to social instead of genetic offspring. In contrast, for females, LRS can be assumed to accurately reflect genetic reproductive output, as intra‐specific brood parasitism in this species is negligible (Turner 2006; Costanzo, Ambrosini, et al. 2017).

Individuals included in the study were selected according to their sex, realized lifespan and cohort. Specifically, we included breeding adults recruited between 2013 and 2015 (i.e., cohorts between 2012 and 2014) that reached different lifespans, trying to balance the number of individuals between sexes, lifespan classes and years. In total, we relied on data from 106 individuals (59 males and 47 females): 14 males and 13 females with lifespan of 1 year, 14 males and 11 females with lifespan of 2 years, 14 males and 9 females with lifespan of 3 years, 9 males and 10 females with lifespan of 4 years, 6 males and 2 females with lifespan of 5 years, 1 male and 2 females with lifespan of 6 years and another male with lifespan of 7 years. While lifespan was known for all individuals, LRS was available for a subset of 76 individuals (35 males and 41 females), because LRS can be estimated only when seasonal reproductive success is recorded for every breeding season. Therefore, for instance, in a breeder reaching a lifespan of 4 years, even a single unassigned clutch in one breeding season prevents us from distinguishing between true non‐breeding and missing observation, and the individual was therefore excluded from LRS analysis. Minor changes in sample size of different analyses (see below) reflect missing or uncollectable data (e.g., broken tail feathers).

### 2.3 Ethics Statement

All capture, blood sampling and marking procedures were performed by experienced bird ringers in accordance with the ASAB/ABS Guidelines (ASAB Ethical Committee/ABS Animal Care Committee, 2023) and under authorization from Regione Lombardia (Decreti No. 329, 21/01/2009; No. 2141, 09/03/2011; No. 2959, 05/04/2012; No. 11316, 01/12/2014; No. 599, 19/01/2018; No. 3268, 12/03/2019).

### 2.4 Spectrometric Ventral Plumage Colour Analysis

Ventral plumage colouration was assessed by measuring the reflectance spectrum of the distal portion of a single feather, as performed by Saino, Romano, et al. (2013). We relied on the tetrahedral colour space model developed for birds to analyse reflectance data (Stoddard and Prum 2008). The model allowed colour to be described in terms of three components: *θ*, which represents the chromatic variation within human visible range, *φ*, which account for the ultraviolet component, and rA which account for colour saturation. Among these variables, only *θ* was retained in the analyses, because *φ* and rA are correlated, colours in the visible spectrum are easier to interpret, and *θ* has been previously associated with telomere length in the barn swallow (Romano et al. 2015; Costanzo, Parolini, et al. 2017; Parolini et al. 2017). In this species, higher *θ* values correspond to paler, more whitish ventral plumage (Saino, Romano, et al. 2013). This method provides highly repeatable measures both between successive measures of the same feather and between measures of different feathers collected from the same ventral region (Saino, Romano, et al. 2013; Romano et al. 2015). Moreover, colouration measured according to this method correlates with measurements performed on live birds, supporting its accuracy as a proxy of in vivo colouration (Romano et al. 2015).

### 2.5 Telomere Length Analysis

We extracted genomic DNA from 10 to 15 μL of red blood cells, with the Wizard DNA extraction kit (Promega, WI, USA). DNA concentration and purity were assessed using a Nanophotometer (NanoDrop One—Thermo Scientific) and we considered as adequate samples with absorbance ratios OD260/280 = 1.8–2.0 and OD260/230 = 2.0–2.2. Telomere length was quantified via monochrome multiplex quantitative PCR method (MMQPCR; Cawthon 2009), according to the protocol developed on the barn swallow by Parolini et al. (2015). Briefly, amplifications were performed on a CFX Opus 96 thermocycler (Bio‐Rad) using a reaction solution composed of 20 ng of template DNA (genomic DNA), 2× Quantitative Master Mix SYBR Green (Genespin) and primers pairs targeting telomere and single copy gene CTCF, at the final concentration of 1000 and 500 nM, respectively. The telomere primers were: telg (5′‐ACACTAAGGTTTGGGTTTGGGTTTGGGTTTGGGTTAGTGT‐3′) and telc (5′‐TGTTAGGTATCCCTATCCCTATCCCTATCCCTATCCCTAACA‐3′). The CTCF primers were: forward (5′‐CCCGCGGCGGGCGGCGCGGGCTGGGCGGCTCCCAATGGAGACCTCAC‐3′) and reverse (5′‐CGCCGCGGCCCGCCGCGCCCGTCCCGCCCATCACCGGTCCATCATGC‐3′). CTCF primers include a GC‐clamp (underlined parts) to enable the amplification of both target sequences in the same reaction well. A four‐point standard curve (5, 10, 20 and 50 ng of a reference DNA sample) was included in each plate to calculate reaction efficiency, using the formula *E* = [10^(−1/*a*)^ − 1] × 100 with *a* representing the slope of the standard curve, and the amount of telomeric and CTCF repeats in each sample. All the samples and the four dilutions of the standard curve were run in triplicate. Due to mismatches in the primer sequences relative to a TTAGGG repeat (Parolini et al. 2018), MMQPCR usually yields lower efficiencies for telomere amplification than conventional qPCR. Therefore, relative telomere length (hereafter, RTL) was estimated according to the Pfaffl formula (Pfaffl 2001), which accounts for differences in amplification efficiency between telomere and CTCF reactions.

Mean (±SD) amplification efficiencies were 132% ± 22% for telomeres and 99% ± 16% for CTCF. The mean intra‐ and inter‐plate coefficients of variation were 4.61% and 3.45%, respectively.

Among the 106 individuals included in the analyses, RTL was measured in all years of life (from recruitment to death) for 89 individuals (27 with lifespan of 1 year, i.e., not surviving to the second year), while 17 individuals lack RTL measures in at least 1 year of life (overall, 23 RTL measures), because of missing/unsuitable DNA samples (e.g., not collected, missing or used previously for other purposes) or problems during RTL quantification. Consequently, 106 individuals resulted in 264 RTL measures collected between 2013 and 2018, with the maximum age at which RTL was available corresponding to 5‐year‐old.

RTL was log‐transformed (log_e_) prior to analyses to improve the normality of its distribution and reduce right‐skewness of qPCR estimates (Nettle et al. 2019), following the same procedure used by several previous studies on other species (e.g., Zee et al. 2010; Huzen et al. 2014; Lynch et al. 2016), including birds (e.g., Reichert et al. 2017; Bauer et al. 2018; Xiong et al. 2025). Therefore, in the ‘Sections 2.6 and 3’ (see below), whenever RTL is used as a dependent or an independent variable, it refers to log‐transformed (log_e_) RTL.

Furthermore, as a measure of RTL attrition rate, we calculated the annual variation in RTL (RTL year_*i*_ − RTL year_*i*−1_; hereafter, ΔRTL). Therefore, ΔRTL values included in our analyses were calculated only between measurements from two consecutive years. To control for the regression to the mean effect, the statistical artefact arising from the correlation between the change in two successive measurements and the baseline measure (here: *r* = −0.63, *t*_154_ = −10.16, *p* < 0.001), we corrected all ΔRTL values included in the analyses following the method proposed by Verhulst et al. (2013). This approach adjusts each observed ΔRTL by subtracting the deviation expected from the regression to the mean, estimated from the correlation between two successive measurements.

### 2.6 Statistical Analyses

To investigate whether RTL shortens with age over the individual lifespan, we fitted a linear mixed‐effects model (LMM), including RTL as a dependent variable (*N* = 264 datapoints, 106 individuals). Fixed effects were age and sex, while individual identity (ID) was modelled as a random intercept to account for repeated measures of individuals. Moreover, we also tested the interaction between sex and age to investigate whether the potential effect of age on RTL differs between males and females. Furthermore, to disentangle between‐ and within‐individual effects of age, we fitted the same LMM while partitioning age into age_mean_ (mean of available ages of an individual in the sample) and age_dev_ (actual age of that individual at a given measure − age_mean_ of the same individual). All these LMMs were also refitted using annual ΔRTL, *z*‐transformed to improve model residuals distribution, as a dependent variable (*N* = 156 datapoints, 78 individuals). In these cases, age refers to the age of the individual at the most recent sampling year relative to each annual ΔRTL datum (i.e., ΔRTL between 1‐ and 2‐year‐old: age = 2‐year‐old; ΔRTL between 2‐ and 3‐year‐old: age = 3‐year‐old; and similarly for subsequent age intervals).

To investigate the association between telomere length and lifespan, we relied on a mixed‐effects Cox proportional‐hazard model (*N* = 106). Lifespan was included as a dependent variable, while RTL at recruitment, sex and both tail length and ventral plumage colouration (hereafter, *θ*) at recruitment (both mean‐centred within sex) were included as fixed effects. Furthermore, we also tested the interaction between RTL at recruitment and sex. The cohort (2012–2014) was included as a random intercept to account for potential variability in survival between years.

Additionally, we investigated the possible association between RTL at recruitment and LRS by fitting a generalized linear mixed model (GLMM) assuming a negative binomial distribution to account for data overdispersion (*N* = 76). LRS was included as a dependent variable with RTL at recruitment, sex and both tail length and *θ* at recruitment (both mean‐centred within sex) as fixed effects. Cohort was included as a random intercept. Also in this case, we tested for the effect of the interaction between RTL at recruitment and sex.

To test whether and how RTL is associated with secondary sexual traits expression, we fitted a LMM (*N* = 254 datapoints, 104 individuals) including RTL as a dependent variable and tail length, *θ*, sex and age, as fixed effects. Tail length and *θ* were mean‐centred within age class (0 = yearling's measures; 1 = 2‐ or more‐year‐old's measures) and sex, to account for the known variation in these traits between yearlings and older adults as well as between males and females (e.g., Saino et al. 2004, 2017; Turner 2006; Saino, Romano, et al. 2013; Romano, Costanzo, et al. 2017). Random grouping factors were year (2013–2018) and individual ID to control for inter‐annual and inter‐individual variability. Moreover, we fitted another LMM with annual ΔRTL as the response variable (*N* = 148 datapoints, 76 individuals). Predictors included annual variation in tail length (tail length year_*i*_ − tail length year_*i*−1_), annual variation in *θ* (*θ* year_*i*_ − *θ* year_*i*−1_), sex and age. Again, year and individual ID were included as random grouping factors. To test for sex‐specific patterns, we also included the interaction terms between sex and the other terms, except for age, in both models.

All statistical analyses were implemented in R version 4.5.1 (R Core Team 2024). LMMs were fitted using the *lmer* function from the ‘lme4’ package (Bates et al. 2015). The mixed effects Cox proportional hazard model was fitted using the *coxme* function from the ‘coxme’ package (Therneau 2024). The GLMM was fitted using the *glmmTMB* function of the package ‘glmmTMB’ (Brooks et al. 2017). We assessed model assumptions for LMMs using the *testResiduals* function of the ‘DHARMa’ package (Hartig 2016), confirming that all LMMs met the assumptions. Additionally, we also found no evidence of influential outliers or multicollinearity, as indicated by the functions *check_outliers* and *check_collinearity* from the ‘performance’ package (Lüdecke et al. 2021), respectively. Overdispersion in the GLMM was assessed using the *check_overdispersion* function of the package ‘performance’ (Lüdecke et al. 2021). Interaction terms were retained in the final models only when statistically significant.

## 3 Results

### 3.1 Age‐Related Change in Telomere Length

**FIGURE 1:**
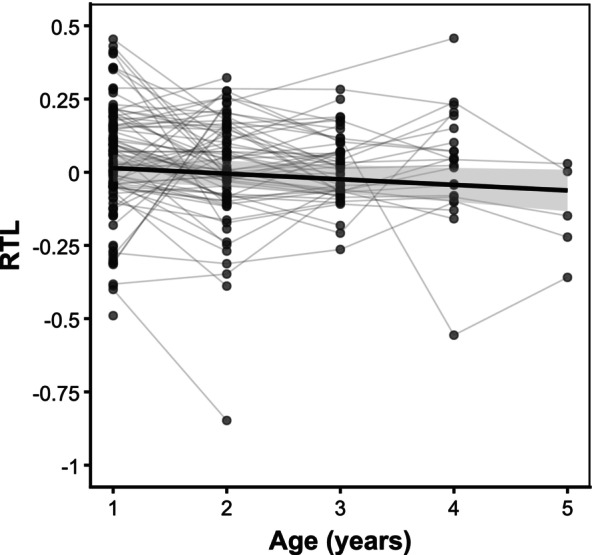
Relationship between individual age and log_e_‐transformed RTL. The black line represents the effect of age on RTL estimated by the linear mixed model reported in Table 1. The grey shaded band represents the 95% confidence interval around the model estimates. Points represent the observed values of the relationship between age and RTL. Light grey lines connect repeated within‐individual RTL measurements at different ages.

RTL progressively shortened with age (Table 1; Figure 1) and did not differ between the sexes (Table 1). This age‐related shortening occurred in a similar way between males and females, as the interaction between age and sex did not reach statistical significance (*χ* ^2^ = 0.86, df = 1, *p* = 0.35). Furthermore, this pattern was mainly driven by the within‐individual variation component of age (age_dev_: *β* = −0.03 ± 0.01 SE, *t*_145.38_ = −2.82, *p* = 0.005; age_mean_: *β* = 0.03 ± 0.02 SE, *t*_92.95_ = 1.44, *p* = 0.15). Additionally, ΔRTL did not vary during lifespan and between sexes, because age (Table 1), as well as both age_mean_ and age_dev_ (age_dev_: *β* = 0.007 ± 0.117 SE, *t*_152_ = 0.06, *p* = 0.95; age_mean_: *β* = 0.12 ± 0.18 SE, *t*_152_ = 0.66, *p* = 0.51) and sex (Table 1) did not significantly predict ΔRTL. There was no age‐related pattern of RTL variation in either males or females, as the interaction between age and sex was not statistically significant (*χ* ^2^ = 0.07, df = 1, *p* = 0.79).

**TABLE 1:** Summaries of the linear mixed models assessing the variation in log_e_‐transformed RTL and ΔRTL (*z*‐transformed) according to age and sex.

RTL				
Predictors	Estimate	CI	df	*p*
Intercept	0.03	−0.02 to 0.09	259	0.23
Age	**−0.02**	**−0.04 to −0.01**	259	**0.036**
Sex (female)	0.03	−0.03 to 0.09	259	0.33
**Random effects**	**τ_00_ (SE)**			
Individual ID	0.01 (0.11)			

ΔRTL				
Predictors	Estimate	CI	df	*p*
Intercept	−0.19	−0.77 to 0.39	151	0.51
Age	0.04	−0.15 to 0.23	151	0.67
Sex (female)	0.14	−0.18 to 0.46	151	0.38
**Random effects**	**τ_00_ (SE)**			
Individual ID	< 0.001 (< 0.001)			

*Note:* In both models, individual ID was included as a random intercept. Statistically significant effects (P < 0.05) are reported in bold.

### 3.2 Telomere Length, Lifespan and Lifetime Reproductive Success

**FIGURE 2:**
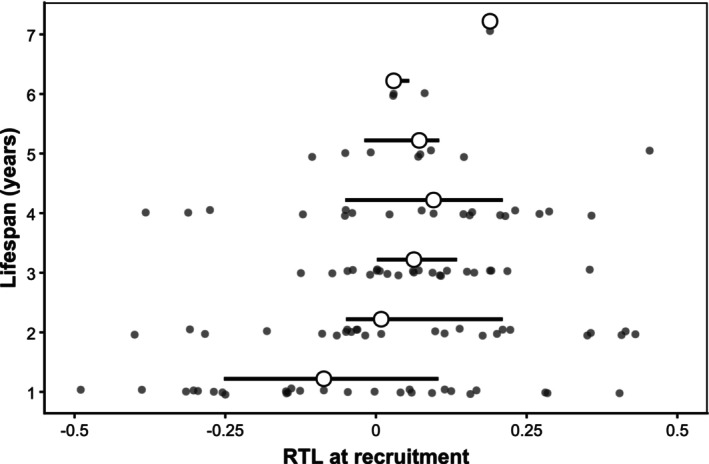
Distribution of log_e_‐transformed RTL at recruitment across lifespan classes. Black points represent the observed individual values. Open circles and horizontal bars represent the median and the interquartile range (Q1–Q3) of RTL within each lifespan class.

Lifespan was positively predicted by RTL at recruitment (Table 2, Figure 2), indicating that individuals with longer telomeres at first reproduction lived significantly longer (Hazard ratio: 0.29). No statistically significant effects emerged for any other predictor (Table 2). The interaction between RTL at recruitment and sex was also not significant (*χ* ^2^ = 0.06, df = 1, *p* = 0.80), meaning that the positive association between RTL and lifespan was similar in males and females.

**TABLE 2:** Summaries of the models assessing whether log_e_‐transformed RTL at recruitment predicted realized lifespan and lifetime reproductive success, while controlling for sex, tail length and ventral plumage colouration at recruitment.

Lifespan				
Predictors	Coefficient	SE	*z*	*p*
RTL at recruitment	**−1.24**	0.60	−2.05	**0.041**
Sex (female)	−0.29	0.21	−1.40	0.16
Tail length at recruitment^a^	0.02	0.01	1.60	0.11
Ventral plumage colouration (*θ*) at recruitment^a^	0.32	1.84	0.18	0.86
**Random effects**	**τ_00_ (SE)**			
Cohort	0.05 (0.22)			

Lifetime reproductive success				
Predictors	Estimate	CI	*z*	*p*
Intercept	**2.70**	**2.48 to 2.91**	24.14	**< 0.001**
RTL at recruitment	**1.23**	**0.31 to 2.15**	2.61	**0.009**
Sex (female)	−0.31	−0.63 to 0.01	−1.85	0.051
Tail length at recruitment^a^	−0.01	−0.04 to 0.01	−0.94	0.35
Ventral plumage colouration (*θ*) at recruitment^a^	−0.76	−3.50 to 1.98	−0.55	0.58
**Random effects**	**τ_00_ (SE)**			
Cohort	< 0.001 (< 0.001)			

*Note:* To test lifespan, we relied on a mixed‐effects Cox proportional hazard model, whereas for lifetime reproductive success, on negative binomial mixed model. Cohort was included as a random intercept in both models. Statistically significant effects (P < 0.05) are reported in bold in all models.

^a^ Mean‐centred within sex to account for well‐known differences between males and females.

**FIGURE 3:**
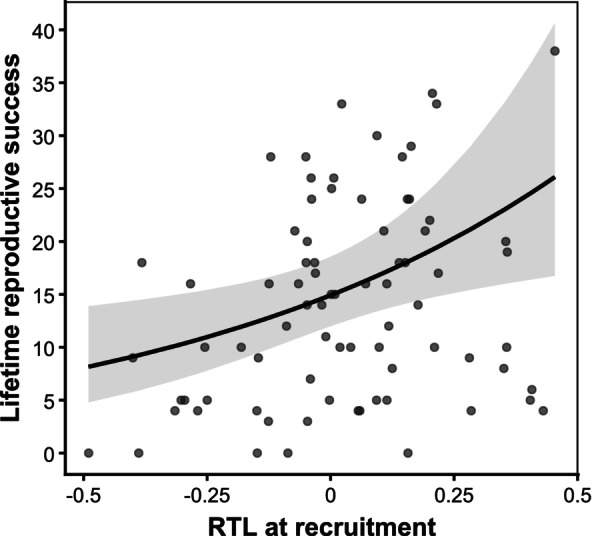
Relationships between log_e_‐transformed RTL at recruitment and lifetime reproductive success (number of fledglings produced during the entire lifespan). Points represent the observed values, while the curve represents the prediction of the negative binomial mixed model reported in Table 2. Shaded area represents the upper and lower limits of the 95% confidence interval of the fitted function.

RTL at recruitment was also positively associated with LRS (Table 2; Figure 3). Sex, tail length and *θ* at recruitment did not reach statistical significance (Table 2). Similarly, the interaction between sex and RTL at recruitment was not statistically significant (*χ* ^2^ = 0.30, df = 1, *p* = 0.58).

To assess whether this positive association was mainly driven by lifespan or also by a higher average reproductive rate of individuals starting adulthood with longer telomeres, we fitted a LMM testing the possible association between RTL at recruitment and the mean number of fledglings produced per breeding season, including sex of the individuals as a dummy variable and cohort as a random intercept. Despite a positive trend emerging, RTL at recruitment did not significantly predict average seasonal reproductive output (RTL at recruitment: *β* ± SE = 1.94 ± 1.38, *t*_75.93_ = 1.40, *p* = 0.16; Sex (female): *β* ± SE = −0.99 ± 0.55, *t*_74.97_ = −1.79, *p* = 0.07), suggesting that its association with LRS is mainly driven by lifespan.

### 3.3 Telomere Length and Expression of Plumage Ornaments

**FIGURE 4:**
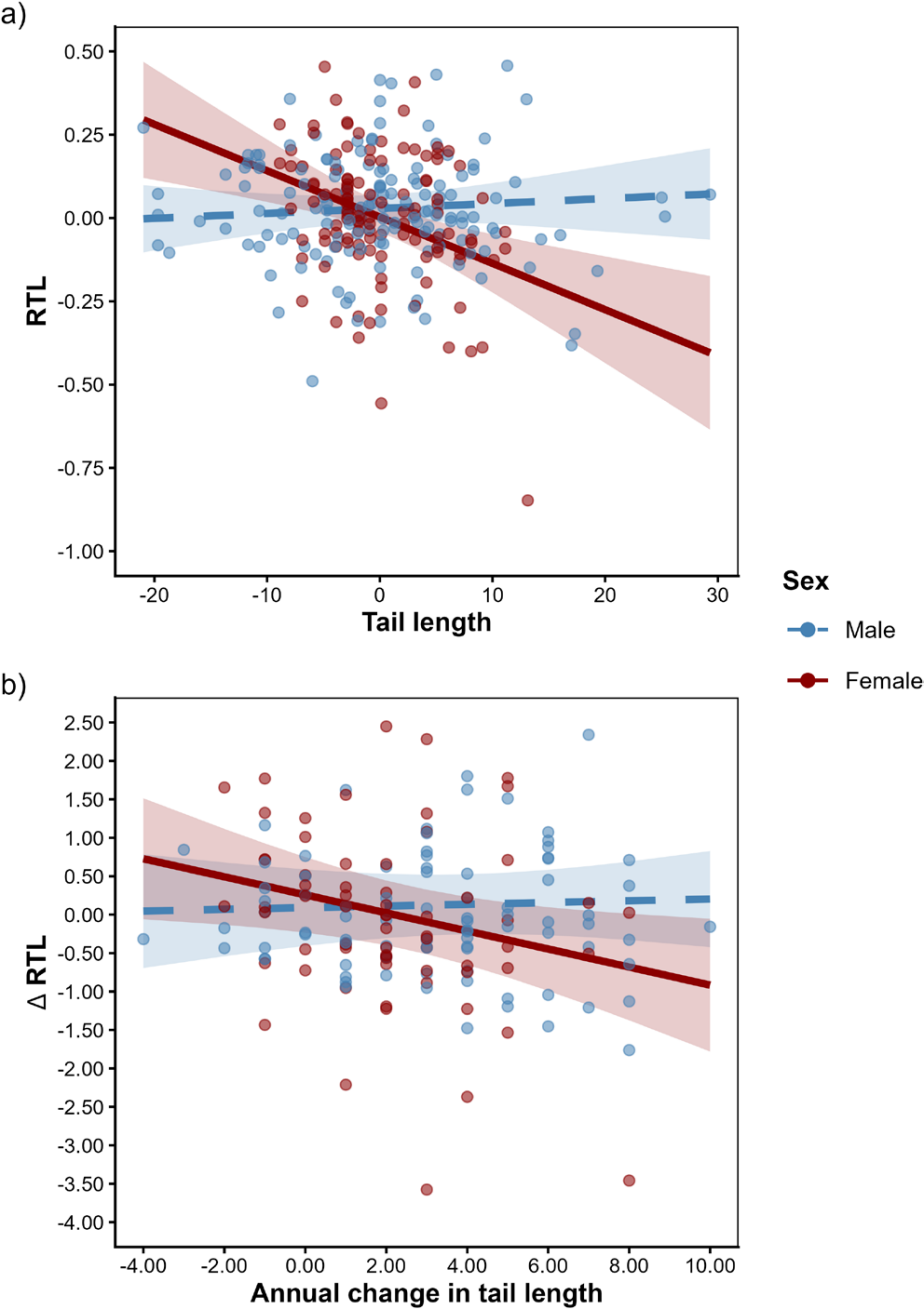
(a) Relationships between outermost tail feathers length (mean centred within sex and age class: 0 = 1‐year‐old; 1 = ≥ 2‐year‐old) and log_e_‐transformed RTL in the two sexes; (b) associations between annual change in tail length and ΔRTL (*z*‐transformed) in the two sexes. In both plots, solid and dashed lines represent statistically significant and non‐statistically significant effects within each sex estimated by the models in Table 3, respectively, while shaded bands illustrate the relative 95% confidence interval.

The interaction between sex and tail length showed a statistically significant effect on RTL (Table 3, Figure 4), indicating that the relationship between tail length and RTL differed between males and females. Specifically, in females, longer tails were associated with shorter RTL (*β* = −0.014 ± 0.004, *t*_151_ = −3.47, *p* < 0.001), whereas no significant association emerged in males (*β* = 0.001 ± 0.002, *t*_106_ = 0.65, *p* = 0.52). No other predictor reached statistical significance (Table 3), and the same was true for the interaction between sex and *θ* (*χ* ^2^ = 0.66, df = 1, *p* = 0.41).

**TABLE 3:** Summaries of the linear mixed models assessing: Whether log_e_‐transformed RTL was associated with tail length, ventral plumage colouration (*θ*), age, sex and the interaction between sex and tail length; and whether ΔRTL (*z*‐transformed) was associated with annual changes in tail length and *θ*, age, sex and the interaction between sex and annual change in tail length.

RTL				
Predictors	Estimate	CI	df	*p*
Intercept	0.04	−0.02 to 0.10	245	0.22
Tail length^a^	**−0.01**	**−0.02 to −0.01**	245	**< 0.001**
Ventral plumage colouration (*θ*)^a^	−0.26	−0.66 to 0.14	245	0.21
Age	−0.02	−0.040 to −0.001	245	0.07
Sex (female)	0.03	−0.03 to 0.08	245	0.33
Sex*Tail length^a^	**0.02**	**0.01 to 0.02**	245	**0.001**
**Random effects**	**τ_00_ (SE)**			
Individual ID	0.01 (0.11)			
Year	< 0.001 (0.02)			

ΔRTL				
Predictors	Estimate	CI	df	*p*
Intercept	0.17	−0.70 to 1.03	139	0.70
Annual change in ventral plumage colouration (*θ*)	**−3.53**	**−6.53 to −0.53**	139	**0.021**
Annual change in tail length	**−0.12**	**−0.22 to −0.02**	139	**0.024**
Age	−0.02	−0.27 to 0.23	139	0.88
Sex (female)	−0.17	−0.62 to 0.28	139	0.47
Sex*Annual change in tail length	**0.13**	**0.01 to 0.25**	139	**0.037**
**Random effects**	**τ_00_ (SE)**			
Individual ID	< 0.001 (< 0.001)			
Year	0.09 (0.29)			

*Note:* Individual ID and year were included as random intercepts in both models. Statistically significant effects (P < 0.05) are reported in bold in all models.

^a^ Mean‐centred within age class (0 = measures at recruitment; 1 = measures in ages ≥ 2 years) and sex due to the known differences between both age classes and sexes.

**FIGURE 5:**
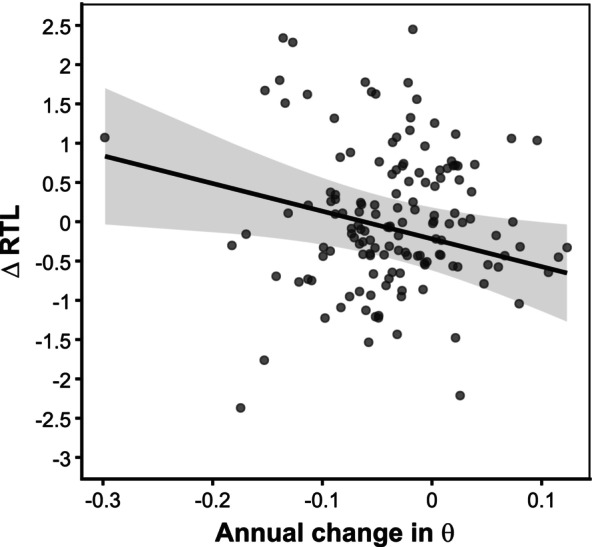
Relationship between annual change in ventral plumage colouration (*θ*) and ΔRTL (*z*‐transformed). Points represent individual observations, while the line represents the fitted values from the linear mixed model reported in Table 3. The shaded band indicates the 95% confidence interval around the model prediction. Increasing values of annual change in *θ* indicate progressive plumage lightening, whereas decreasing values indicate progressive plumage darkening.

Consistent with the pattern observed for RTL, the LMM on ΔRTL showed a statistically significant effect of the interaction between sex and annual change in tail length (Table 3; Figure 4). In females, a larger increase in tail length between consecutive years was associated with faster telomere shortening over the same timespan (*β* = −0.12 ± 0.05, *t*_133_ = −2.25, *p* = 0.026), whereas this relationship was not significant in males (*β* = 0.01 ± 0.04, *t*_133_ = 0.27, *p* = 0.78). Furthermore, ΔRTL was predicted by annual change in *θ*, with plumage darkening between consecutive years corresponding to reduced telomere shortening over the same interval (Table 3; Figure 5). Notably, this pattern held in both sexes, as the interaction between annual changes in *θ* and sex was statistically non‐significant (*χ* ^2^ = 0.02, df = 1, *p* = 0.89). Other predictors did not show a statistically significant association with ΔRTL (Table 3).

## 4 Discussion

Telomere length and dynamics have been suggested as key predictors of inter‐individual variation in life‐history traits, with telomere length potentially reflecting individual somatic state, and telomere attrition rate reflecting the efficiency of mechanisms underlying somatic maintenance (Haussmann and Marchetto 2010). In this longitudinal study of a short‐lived migratory passerine, we confirmed the age‐related decline in telomere length reported across vertebrates and found that telomeres are linked to major fitness components as individuals with longer telomeres at recruitment lived longer and achieved higher LRS. In addition, telomere length and dynamics covaried with sexually selected traits. In females, longer tails and stronger increases in tail length between consecutive years were associated with shorter telomeres and faster telomere attrition, respectively, whereas in both sexes, ventral plumage darkening between consecutive years was related to reduced telomere attrition over the same time span.

RTL at recruitment (i.e., 1‐year‐old) positively predicted realized lifespan, meaning that individuals with longer telomeres at the beginning of adulthood lived longer. Our result aligns with studies on other wild bird populations reporting positive association between telomere length measured early in life (or at the beginning of adulthood) and long‐term survival/longevity (Eastwood et al. 2019; Bichet et al. 2020; Vedder et al. 2022; Eastwood, Dupoué, Delhey, et al. 2023; Chik et al. 2024), and is therefore consistent with the idea that telomere length can reflect inter‐individual variation in somatic state (Monaghan 2010, 2024). Notably, in the same study population, RTL measured at the nestling stage predicts survival beyond the first year of life (Novelli et al. 2026) and, together with our finding, suggests that differences in somatic state may be established early in life and may persist into recruitment. Two non‐mutually exclusive scenarios may explain this relationship. First, telomeres may contribute causally to survival because critically short telomeres can trigger cellular senescence and apoptosis, thereby impairing tissue renewal capacity and organismal performance (Campisi et al. 2001; Deng et al. 2008). Under this scenario, individuals entering adulthood with longer RTL may require more time to reach the critical thresholds associated with functional decline and increased mortality risk (Monaghan 2010, 2024). This may be further supported by our finding showing that RTL shortens with age at a constant rate, as reported across vertebrate species (Remot et al. 2022). Second, RTL at recruitment may correlate with lifespan simply because both are influenced by shared underlying determinants (e.g., early‐life environment), rather than because telomeres directly cause differences in longevity (Monaghan 2010, 2024; Young 2018). Because our study is correlational, it cannot disentangle these possible pathways. Nonetheless, our result suggests that, even in a short‐lived migratory passerine species where stochastic mortality is expected to be extremely high, individual somatic state may contribute to inter‐individual variation in longevity.

RTL at recruitment also positively predicted LRS, expressed as the total number of fledglings produced over the entire individual life. In iteroparous species, lifespan is a major determinant of LRS because it predicts the number of reproductive events (Newton 1989), and this is also the case for the barn swallow (Saino et al. 2012). Accordingly, as reported in other bird species (e.g., Eastwood et al. 2019; Heidinger et al. 2021), the positive association between RTL at recruitment and LRS is most likely mediated by lifespan, because individuals with longer telomeres at the onset of reproduction lived longer and thus had more opportunities to breed. However, a second and non‐mutually exclusive pathway may also exist. Indeed, telomere length at the beginning of adulthood may predict LRS even when the association with lifespan is not detected, suggesting that individuals starting adulthood with longer telomeres may also achieve higher LRS through consistently higher seasonal reproductive performance (Chik et al. 2024). In our dataset, although the association between RTL at recruitment and the average number of fledglings produced per breeding season was positive, it did not reach statistical significance, indicating that the relationship between RTL at recruitment and LRS is likely to be primarily driven by the link between RTL and lifespan, rather than by higher seasonal reproductive rates. Therefore, our results generally indicate that longer telomeres at the onset of reproduction are associated with higher individual quality, expressed in terms of both higher lifespan and LRS, and may therefore be favoured by natural selection. Because extra‐pair fertilization occurs in this species and in our study population (12.7%; Costanzo, Ambrosini, et al. 2017), male LRS reflects social rather than strictly genetic offspring in a minority of cases, whereas female LRS can be assumed to represent the overall genetic reproductive output. However, since LRS and lifespan are positively correlated (Saino et al. 2012), any bias introduced by extra‐pair paternity of males is unlikely to qualitatively affect the interpretation of the observed patterns.

In addition, telomere length and dynamics across adulthood were associated with the expression of a sexually selected trait in the barn swallow, the length of the outermost tail feathers (Costanzo, Ambrosini, et al. 2017; Romano, Saino, and Møller 2017; Romano, Costanzo, et al. 2017), although the relationships were sex specific. In particular, females with longer tails had shorter RTL, and females that increased tail length more between consecutive years experienced faster telomere shortening over the same interval. These parallel patterns for RTL and ΔRTL highlight the possibility that investment in tail ornamentation can entail somatic costs in females of this species. Similar negative associations between ornamentation and telomere dynamics have been reported in other vertebrates (Azcárate‐García et al. 2020; Kauzálová et al. 2022), including another barn swallow population where tail length covaried negatively with telomere length (Kauzálová et al. 2022). Notably, the sex specificity of our results suggests that the costs and/or benefits of tail feather production may differ between males and females. In barn swallows, tail length is under strong sexual selection in males, and longer tails are more consistently associated with male quality and mating advantages (Saino et al. 1997; Costanzo, Ambrosini, et al. 2017; Romano, Saino, and Møller 2017; Romano, Costanzo, et al. 2017). Females, by contrast, may express relatively elongated streamers partly due to genetic correlation with tail length of the father, even though the trait is not the primary target of sexual selection in females (Cuervo and Møller 2000). When females express more male‐like tail length, they may incur disproportionate physiological costs associated with this male‐typical trait expression (Cuervo 2003; Vitousek et al. 2016), which may lead to faster telomere shortening e.g., (flight costs—Schultner et al. 2014; Bauer et al. 2016; Atema et al. 2021). At the same time, females also bear additional reproductive costs associated with egg production and exclusive incubation (Turner 2006). The combined burden of reproduction and ornament expression could therefore constrain somatic maintenance more strongly in females, leading to detectable telomere costs associated with tail investment.

Interestingly, we also found that annual changes in ventral plumage colouration (*θ*) predicted ΔRTL, with individuals that darkened more between consecutive years showing reduced telomere shortening over the same interval. This relationship held in both sexes, suggesting that the processes linking changes in plumage colouration and telomere dynamics are not sex‐limited, as observed for tail length. This finding complements previous evidence from the same barn swallow population reporting that darker ventral colouration covaries with longer telomeres both in breeding adults (Parolini et al. 2017) and nestlings (Costanzo, Parolini, et al. 2017). Taken together, these results suggest a coherent scenario, whereby darker ventral plumage colouration is associated not only with longer telomeres at a given time, but also with improved telomere maintenance across years, reinforcing the view that telomere dynamics capture biologically meaningful variation in individual quality.

By linking telomere length and dynamics to lifespan, LRS, and sexually selected traits expression within the same longitudinal study, our results show that telomere biology can be informative of inter‐individual variation in fitness‐related traits, even in short‐lived migratory species where very high annual mortality may reduce the strength of the association between somatic state and thus telomere length and lifespan. Specifically, as RTL at recruitment predicts lifespan and LRS, our findings imply that differences in somatic state at the onset of reproduction can translate into considerable fitness differences, making telomere length a plausible target of selection in this population. At the same time, the sex‐specific association between RTL/ΔRTL and tail ornamentation, together with the covariation between annual changes in ventral plumage colouration and ΔRTL, highlights that telomere maintenance can also reveal how investment in secondary sexual traits relates to possible sex‐ and trait‐specific physiological constraints in the study species. Overall, these findings support the idea that telomeres are linked to natural and sexual selection processes in the wild, and highlight the need for both experimental studies manipulating reproductive and ornamental costs and comparative longitudinal work across species and ecological contexts to test whether the relationships between telomeres and fitness mirror species‐specific life histories and may therefore have contributed to their evolution.

## Author Contributions

A.N. and A.R. conceived and designed the study. A.N. carried out laboratory work under the supervision of M.P. and with the assistance of M.C., performed the statistical analyses, and wrote the manuscript. A.R. provided major supervision throughout the statistical analyses and manuscript preparation, participated in fieldwork and revised the manuscript. M.C. assisted with laboratory work and participated in fieldwork. M.P. supervised laboratory work and revised the manuscript. R.A. helped in statistical analyses; R.A., A.C. and D.R. participated in fieldwork and revised the manuscript. All authors approved the final version of the manuscript.

## Funding

The authors have nothing to report.

## Disclosure

Benefit‐Sharing Statement: Benefits from this research accrue from the public sharing of data and results as described above.

## Conflicts of Interest

The authors declare no conflicts of interest.

## Data Availability

The data and R code supporting this study are deposited in Data@UNIMI: https://dataverse.unimi.it/dataset.xhtml?persistentId=doi:10.13130/RD_UNIMI/ILKZH9.

## References

[mec70496-bib-0001] Andersson, M. 1994. Sexual Selection. Princeton University press.

[mec70496-bib-0002] Armstrong, E. , and J. Boonekamp . 2023. “Does Oxidative Stress Shorten Telomeres In Vivo? A Meta‐Analysis.” Ageing Research Reviews 85: 101854. 10.1016/j.arr.2023.101854. 36657619

[mec70496-bib-0099] Atema, E. , A. J. van Noordwijk , and S. Verhulst . 2021. “Telomere Dynamics in Relation to Experimentally Increased Locomotion Costs and Fitness in Great Tits.” Molecular Ecology 31, no. 23: 6208–6215. 10.1111/mec.16162. 34478576 PMC9786264

[mec70496-bib-0003] Azcárate‐García, M. , M. Ruiz‐Rodríguez , S. Díaz‐Lora , et al. 2020. “Ornamental Throat Feathers Predict Telomere Dynamic and Hatching Success in Spotless Starling ( *Sturnus unicolor* ) Males.” Frontiers in Ecology and Evolution 7: 520. 10.3389/fevo.2019.00520.

[mec70496-bib-0004] Badás, E. P. , C. Bauch , J. J. Boonekamp , E. Mulder , and S. Verhulst . 2023. “Ectoparasite Presence and Brood Size Manipulation Interact to Accelerate Telomere Shortening in Nestling Jackdaws.” Molecular Ecology 32, no. 24: 6913–6923. 10.1111/mec.17177. 37864481

[mec70496-bib-0005] Bates, D. , M. Mächler , B. Bolker , and S. Walker . 2015. “Fitting Linear Mixed‐Effects Models Using lme4.” Journal of Statistical Software 67, no. 1: 1–48. 10.18637/jss.v067.i01.

[mec70496-bib-0006] Bauch, C. , J. Riechert , S. Verhulst , and P. H. Becker . 2016. “Telomere Length Reflects Reproductive Effort Indicated by Corticosterone Levels in a Long‐Lived Seabird.” Molecular Ecology 25, no. 22: 5785–5794. 10.1111/mec.13874. 27696588

[mec70496-bib-0007] Bauer, C. M. , J. L. Graham , M. Abolins‐Abols , B. J. Heidinger , E. D. Ketterson , and T. J. Greives . 2018. “Chronological and Biological Age Predict Seasonal Reproductive Timing: An Investigation of Clutch Initiation and Telomeres in Birds of Known Age.” American Naturalist 191, no. 6: 777–782. 10.1086/697224. 29750556

[mec70496-bib-0098] Bauer, C. M. , B. J. Heidinger , E. D. Ketterson , and T. J. Greives . 2016. “A Migratory Lifestyle is Associated With Shorter Telomeres in a Songbird (*Junco hyemalis*).” Auk 133, no. 4: 649–653. 10.1642/auk-16-56.1.

[mec70496-bib-0008] Bichet, C. , S. Bouwhuis , C. Bauch , S. Verhulst , P. H. Becker , and O. Vedder . 2020. “Telomere Length Is Repeatable, Shortens With Age and Reproductive Success, and Predicts Remaining Lifespan in a Long‐Lived Seabird.” Molecular Ecology 29, no. 2: 429–441. 10.1111/mec.15331. 31841253

[mec70496-bib-0009] Blackburn, E. H. 1991. “Structure and Function of Telomeres.” Nature 350, no. 6319: 569–573. 10.1038/350569a0. 1708110

[mec70496-bib-0010] Blackburn, E. H. 2005. “Telomeres and Telomerase: Their Mechanisms of Action and the Effects of Altering Their Functions.” FEBS Letters 579, no. 4: 859–862. 10.1016/j.febslet.2004.11.036. 15680963

[mec70496-bib-0011] Boonekamp, J. J. , G. A. Mulder , H. M. Salomons , C. Dijkstra , and S. Verhulst . 2014. “Nestling Telomere Shortening, but Not Telomere Length, Reflects Developmental Stress and Predicts Survival in Wild Birds.” Proceedings of the Royal Society B: Biological Sciences 281, no. 1785: 20133287. 10.1098/rspb.2013.3287. PMC4024283 24789893

[mec70496-bib-0012] Brooks, M. E. , K. Kristensen , K. J. van Benthem , et al. 2017. “glmmTMB Balances Speed and Flexibility Among Packages for Zero‐Inflated Generalized Linear Mixed Modeling.” R Journal 9, no. 2: 378. 10.32614/RJ-2017-066.

[mec70496-bib-0013] Brown, T. J. , L. G. Spurgin , H. L. Dugdale , J. Komdeur , T. Burke , and D. S. Richardson . 2022. “Causes and Consequences of Telomere Lengthening in a Wild Vertebrate Population.” Molecular Ecology 31, no. 23: 5933–5945. 10.1111/mec.16059. 34219315

[mec70496-bib-0014] Campisi, J. , S. Kim , C.‐S. Lim , and M. Rubio . 2001. “Cellular Senescence, Cancer and Aging: The Telomere Connection.” Experimental Gerontology 36, no. 10: 1619–1637. 10.1016/S0531-5565(01)00160-7. 11672984

[mec70496-bib-0015] Caprioli, M. , M. Romano , A. Romano , et al. 2013. “Nestling Telomere Length Does Not Predict Longevity, but Covaries With Adult Body Size in Wild Barn Swallows.” Biology Letters 9, no. 5: 20130340. 10.1098/rsbl.2013.0340. 23883575 PMC3971670

[mec70496-bib-0016] Cawthon, R. M. 2009. “Telomere Length Measurement by a Novel Monochrome Multiplex Quantitative PCR Method.” Nucleic Acids Research 37, no. 3: e21. 10.1093/nar/gkn1027. 19129229 PMC2647324

[mec70496-bib-0017] Chatelain, M. , S. M. Drobniak , and M. Szulkin . 2020. “The Association Between Stressors and Telomeres in Non‐Human Vertebrates: A Meta‐Analysis.” Ecology Letters 23, no. 2: 381–398. 10.1111/ele.13426. 31773847

[mec70496-bib-0018] Chik, H. Y. J. , M. Mannarelli , N. Dos Remedios , et al. 2024. “Adult Telomere Length Is Positively Correlated With Survival and Lifetime Reproductive Success in a Wild Passerine.” Molecular Ecology 33, no. 15: e17455. 10.1111/mec.17455. 38993011

[mec70496-bib-0019] Costantini, D. 2024. “Oxidative Stress and Reproductive Trade‐Offs: From Courtship to Parental Care.” In The Role of Organismal Oxidative Stress in the Ecology and Life‐History Evolution of Animals, edited by D. Costantini , 323–367. Springer Nature Switzerland. 10.1007/978-3-031-65183-0_9.

[mec70496-bib-0020] Costanzo, A. , R. Ambrosini , M. Caprioli , et al. 2017. “Lifetime Reproductive Success, Selection on Lifespan, and Multiple Sexual Ornaments in Male European Barn Swallows: Breeding Success and Selection in Swallows.” Evolution 71, no. 10: 2457–2468. 10.1111/evo.13312. 28722759

[mec70496-bib-0021] Costanzo, A. , M. Parolini , G. Bazzi , et al. 2017. “Brood Size, Telomere Length, and Parent‐Offspring Color Signaling in Barn Swallows.” Behavioral Ecology 28, no. 1: 204–211. 10.1093/beheco/arw147.

[mec70496-bib-0022] Criscuolo, F. , F. S. Dobson , and Q. Schull . 2021. “The Influence of Phylogeny and Life History on Telomere Lengths and Telomere Rate of Change Among Bird Species: A Meta‐Analysis.” Ecology and Evolution 11, no. 19: 12908–12922. 10.1002/ece3.7931. 34646443 PMC8495793

[mec70496-bib-0023] Cuervo, J. J. 2003. “Experimental Manipulation of Tail Length in Female Barn Swallows ( *Hirundo rustica* ) Affects Their Future Reproductive Success.” Behavioral Ecology 14, no. 4: 451–456. 10.1093/beheco/arg027.

[mec70496-bib-0024] Cuervo, J. J. , and A. P. Møller . 2000. “Sex‐Limited Expression of Ornamental Feathers in Birds.” Behavioral Ecology 11, no. 3: 246–259. 10.1093/beheco/11.3.246.

[mec70496-bib-0025] Deng, Y. , S. S. Chan , and S. Chang . 2008. “Telomere Dysfunction and Tumour Suppression: The Senescence Connection.” Nature Reviews Cancer 8, no. 6: 450–458. 10.1038/nrc2393. 18500246 PMC3688269

[mec70496-bib-0026] Eastwood, J. R. , T. Connallon , K. Delhey , et al. 2022. “Hot and Dry Conditions Predict Shorter Nestling Telomeres in an Endangered Songbird: Implications for Population Persistence.” Proceedings of the National Academy of Sciences of the United States of America 119, no. 25: e2122944119. 10.1073/pnas.2122944119. 35696588 PMC9231487

[mec70496-bib-0027] Eastwood, J. R. , A. Dupoué , K. Delhey , S. Verhulst , A. Cockburn , and A. Peters . 2023. “When Does Early‐Life Telomere Length Predict Survival? A Case Study and Meta‐Analysis.” Molecular Ecology 32, no. 11: 3000–3013. 10.1111/mec.16894. 36811398

[mec70496-bib-0028] Eastwood, J. R. , A. Dupoué , S. Verhulst , A. Cockburn , and A. Peters . 2023. “Cool, Dry Nights and Short Heatwaves During Growth Result in Longer Telomeres in Temperate Songbird Nestlings.” Molecular Ecology 32, no. 19: 5382–5393. 10.1111/mec.17107. 37606092

[mec70496-bib-0029] Eastwood, J. R. , M. L. Hall , N. Teunissen , et al. 2019. “Early‐Life Telomere Length Predicts Lifespan and Lifetime Reproductive Success in a Wild Bird.” Molecular Ecology 28, no. 5: 1127–1137. 10.1111/mec.15002. 30592345

[mec70496-bib-0030] Greider, C. W. , and E. H. Blackburn . 1989. “A Telomeric Sequence in the RNA of Tetrahymena Telomerase Required for Telomere Repeat Synthesis.” Nature 337, no. 6205: 331–337. 10.1038/337331a0. 2463488

[mec70496-bib-0031] Harley, C. B. , A. B. Futcher , and C. W. Greider . 1990. “Telomeres Shorten During Ageing of Human Fibroblasts.” Nature 345, no. 6274: 458–460. 10.1038/345458a0. 2342578

[mec70496-bib-0032] Hartig, F. 2016. “DHARMa: Residual Diagnostics for Hierarchical (Multi‐Level/Mixed) Regression Models.” (p. 0.4.7) [Dataset]. 10.32614/CRAN.package.DHARMa.

[mec70496-bib-0033] Haussmann, M. F. , and N. M. Marchetto . 2010. “Telomeres: Linking Stress and Survival, Ecology and Evolution.” Current Zoology 56, no. 6: 714–727. 10.1093/czoolo/56.6.714.

[mec70496-bib-0034] Heidinger, B. J. , A. C. Kucera , J. D. Kittilson , and D. F. Westneat . 2021. “Longer Telomeres During Early Life Predict Higher Lifetime Reproductive Success in Females but Not Males.” Proceedings of the Royal Society B: Biological Sciences 288, no. 1951: 20210560. 10.1098/rspb.2021.0560. PMC8150037 34034512

[mec70496-bib-0035] Huhta, E. , S. Rytkönen , and T. Solonen . 2003. “Plumage Brightness of Prey Increases Predation Risk: An Among‐Species Comparison.” Ecology 84, no. 7: 1793–1799. 10.1890/0012-9658(2003)084[1793:PBOPIP]2.0.CO;2.

[mec70496-bib-0036] Huzen, J. , L. S. M. Wong , D. J. Van Veldhuisen , et al. 2014. “Telomere Length Loss due to Smoking and Metabolic Traits.” Journal of Internal Medicine 275, no. 2: 155–163. 10.1111/joim.12149. 24118582

[mec70496-bib-0037] Kauzálová, T. , O. Tomášek , E. Mulder , S. Verhulst , and T. Albrecht . 2022. “Telomere Length Is Highly Repeatable and Shorter in Individuals With More Elaborate Sexual Ornamentation in a Short‐Lived Passerine.” Molecular Ecology 31, no. 23: 6172–6183. 10.1111/mec.16397. 35150467

[mec70496-bib-0038] Lingner, J. , T. R. Hughes , A. Shevchenko , M. Mann , V. Lundblad , and T. R. Cech . 1997. “Reverse Transcriptase Motifs in the Catalytic Subunit of Telomerase.” Science 276, no. 5312: 561–567. 10.1126/science.276.5312.561. 9110970

[mec70496-bib-0100] Lüdecke, D. , M. Ben‐Shachar , I. Patil , P. Waggoner , and D. Makowski . 2021. “performance: An R Package for Assessment, Comparison and Testing of Statistical Models.” Journal of Open Source Software 6, no. 60: 3139. 10.21105/joss.03139.

[mec70496-bib-0039] Lynch, S. M. , M. K. Peek , N. Mitra , et al. 2016. “Race, Ethnicity, Psychosocial Factors, and Telomere Length in a Multicenter Setting.” PLoS One 11, no. 1: e0146723. 10.1371/journal.pone.0146723. 26752285 PMC4709232

[mec70496-bib-0040] McLennan, D. , J. D. Armstrong , D. C. Stewart , et al. 2018. “Telomere Elongation During Early Development Is Independent of Environmental Temperatures in Atlantic Salmon.” Journal of Experimental Biology 221, no. 11: jeb178616. 29636409 10.1242/jeb.178616 PMC6031317

[mec70496-bib-0041] Merkling, T. , P. Blanchard , O. Chastel , et al. 2017. “Reproductive Effort and Oxidative Stress: Effects of Offspring Sex and Number on the Physiological State of a Long‐Lived Bird.” Functional Ecology 31, no. 6: 1201–1209. 10.1111/1365-2435.12829.

[mec70496-bib-0042] Mizutani, Y. , N. Tomita , Y. Niizuma , and K. Yoda . 2013. “Environmental Perturbations Influence Telomere Dynamics in Long‐Lived Birds in Their Natural Habitat.” Biology Letters 9, no. 5: 20130511. 10.1098/rsbl.2013.0511. 23945210 PMC3971690

[mec70496-bib-0043] Møller, A. P. 1991. “Sexual Selection in the Monogamous Barn Swallow ( *Hirundo rustica* ). I. Determinants of Tail Ornament Size.” Evolution 45, no. 8: 1823–1836. 10.1111/j.1558-5646.1991.tb02690.x. 28563968

[mec70496-bib-0044] Møller, A. P. , C. Magnhagen , A. Ulfstrand , and S. Ulfstrand . 1995. “Phenotypic Quality and Molt in the Barn Swallow, *Hirundo rustica* .” Behavioral Ecology 6, no. 3: 242–249. 10.1093/beheco/6.3.242.

[mec70496-bib-0045] Monaghan, P. 2010. “Telomeres and Life Histories: The Long and the Short of It.” Annals of the New York Academy of Sciences 1206, no. 1: 130–142. 10.1111/j.1749-6632.2010.05705.x. 20860686

[mec70496-bib-0046] Monaghan, P. 2014. “Organismal Stress, Telomeres and Life Histories.” Journal of Experimental Biology 217, no. 1: 57–66. 10.1242/jeb.090043. 24353204

[mec70496-bib-0047] Monaghan, P. 2024. “Linking Telomere Dynamics to Evolution, Life History and Environmental Change: Perspectives, Predictions and Problems.” Biogerontology 25, no. 2: 301–311. 10.1007/s10522-023-10081-8. 38252370 PMC10998769

[mec70496-bib-0048] Monaghan, P. , M. Olsson , D. S. Richardson , S. Verhulst , and S. M. Rogers . 2022. “Integrating Telomere Biology Into the Ecology and Evolution of Natural Populations: Progress and Prospects.” Molecular Ecology 31, no. 23: 5909–5916. 10.1111/mec.16768. 36330668

[mec70496-bib-0049] Monaghan, P. , and S. E. Ozanne . 2018. “Somatic Growth and Telomere Dynamics in Vertebrates: Relationships, Mechanisms and Consequences.” Philosophical Transactions of the Royal Society, B: Biological Sciences 373, no. 1741: 20160446. 10.1098/rstb.2016.0446. PMC5784066 29335370

[mec70496-bib-0050] Morland, F. , J. G. Ewen , M. J. P. Simons , P. Brekke , and N. Hemmings . 2023. “Early‐Life Telomere Length Predicts Life‐History Strategy and Reproductive Senescence in a Threatened Wild Songbird.” Molecular Ecology 32, no. 14: 4031–4043. 10.1111/mec.16981. 37173827 PMC10947174

[mec70496-bib-0051] Morosinotto, C. , S. Bensch , M. Tarka , and P. Karell . 2022. “Heritability and Parental Effects in Telomere Length in a Color Polymorphic Long‐Lived Bird.” Physiological and Biochemical Zoology 95, no. 4: 350–364. 10.1086/720161. 35659559

[mec70496-bib-0052] Nettle, D. , L. Seeker , D. Nussey , H. Froy , and M. Bateson . 2019. “Consequences of Measurement Error in qPCR Telomere Data: A Simulation Study.” PLoS One 14, no. 5: e0216118. 10.1371/journal.pone.0216118. 31042766 PMC6493763

[mec70496-bib-0053] Newton, I. , ed. 1989. Lifetime Reproduction in Birds. Academic Press.

[mec70496-bib-0054] Noguera, J. C. 2017. “Interacting Effects of Early Dietary Conditions and Reproductive Effort on the Oxidative Costs of Reproduction.” PeerJ 5: e3094. 10.7717/peerj.3094. 28316895 PMC5354074

[mec70496-bib-0055] Noguera, J. C. , N. B. Metcalfe , W. Boner , and P. Monaghan . 2015. “Sex‐Dependent Effects of Nutrition on Telomere Dynamics in Zebra Finches ( *Taeniopygia guttata* ).” Biology Letters 11, no. 2: 20140938. 10.1098/rsbl.2014.0938. 25716087 PMC4360105

[mec70496-bib-0056] Nolazco, S. , K. Delhey , S. Nakagawa , and A. Peters . 2022. “Ornaments Are Equally Informative in Male and Female Birds.” Nature Communications 13, no. 1: 5917. 10.1038/s41467-022-33548-7. PMC9546859 36207296

[mec70496-bib-0103] Novelli, A. , M. Caprioli , R. Ambrosini , et al. 2026. “Early‐Life Telomere Length Negatively Covaries With Brood Size and Father Age, and Predicts Long‐Term Survival in Male Barn Swallows (*Hirundo rustica*).” Integrative Zoology, 1–13. 10.1111/1749-4877.70173. 42663320

[mec70496-bib-0057] Olsson, M. , E. Miller , N. Rollings , et al. 2025. “The Effects of Costly Telomere Maintenance on Lifespan: Reproductive Tradeoffs in Sand Lizards.” Evolution 79, no. 5: 847–857. 39688874 10.1093/evolut/qpae181

[mec70496-bib-0058] Parolini, M. , C. D. Possenti , A. Romano , M. Caprioli , D. Rubolini , and N. Saino . 2018. “Physiological Increase of Yolk Testosterone Level Does Not Affect Oxidative Status and Telomere Length in Gull Hatchlings.” PLoS One 13, no. 10: e0206503. 10.1371/journal.pone.0206503. 30365552 PMC6203383

[mec70496-bib-0059] Parolini, M. , A. Romano , A. Costanzo , et al. 2017. “Telomere Length Is Reflected by Plumage Coloration and Predicts Seasonal Reproductive Success in the Barn Swallow.” Molecular Ecology 26, no. 21: 6100–6109. 10.1111/mec.14340. 28851004

[mec70496-bib-0060] Parolini, M. , A. Romano , L. Khoriauli , et al. 2015. “Early‐Life Telomere Dynamics Differ Between the Sexes and Predict Growth in the Barn Swallow ( *Hirundo rustica* ).” PLoS One 10, no. 11: e0142530. 10.1371/journal.pone.0142530. 26565632 PMC4643985

[mec70496-bib-0061] Pauliny, A. , R. H. Wagner , J. Augustin , T. Szép , and D. Blomqvist . 2006. “Age‐Independent Telomere Length Predicts Fitness in Two Bird Species.” Molecular Ecology 15, no. 6: 1681–1687. 10.1111/j.1365-294X.2006.02862.x. 16629820

[mec70496-bib-0062] Pepke, M. L. , T. Kvalnes , P. S. Ranke , et al. 2022. “Causes and Consequences of Variation in Early‐Life Telomere Length in a Bird Metapopulation.” Ecology and Evolution 12, no. 8: e9144. 10.1002/ece3.9144. 35923948 PMC9339764

[mec70496-bib-0063] Pepke, M. L. , T. H. Ringsby , and D. T. A. Eisenberg . 2023. “The Evolution of Early‐Life Telomere Length, Pace‐of‐Life and Telomere‐Chromosome Length Dynamics in Birds.” Molecular Ecology 32, no. 11: 2898–2912. 10.1111/mec.16907. 36847070

[mec70496-bib-0064] Pfaffl, M. W. 2001. “A New Mathematical Model for Relative Quantification in Real‐Time RT‐PCR.” Nucleic Acids Research 29, no. 9: 45e–445e. 10.1093/nar/29.9.e45. PMC55695 11328886

[mec70496-bib-0102] R Core Team . 2024. “R: A Language and Environment for Statistical Computing.” R Foundation for Statistical Computing, Vienna, Austria. https://www.R‐project.org/.

[mec70496-bib-0065] Reichert, S. , H. Froy , W. Boner , et al. 2017. “Telomere Length Measurement by qPCR in Birds Is Affected by Storage Method of Blood Samples.” Oecologia 184, no. 2: 341–350. 10.1007/s00442-017-3887-3. 28547179 PMC5487852

[mec70496-bib-0066] Remot, F. , V. Ronget , H. Froy , et al. 2022. “Decline in Telomere Length With Increasing Age Across Nonhuman Vertebrates: A Meta‐Analysis.” Molecular Ecology 31, no. 23: 5917–5932. 10.1111/mec.16145. 34437736

[mec70496-bib-0067] Romano, A. , R. Ambrosini , M. Caprioli , A. Costanzo , A. Novelli , and D. Rubolini . 2025. “Shrinking Body Size Under Climate Warming Is Not Associated With Selection for Smaller Individuals in a Migratory Bird.” Journal of Animal Ecology 94, no. 5: 958–970. 10.1111/1365-2656.70027. 40176260 PMC12056357

[mec70496-bib-0068] Romano, A. , A. Costanzo , D. Rubolini , N. Saino , and A. P. Møller . 2017. “Geographical and Seasonal Variation in the Intensity of Sexual Selection in the Barn Swallow *Hirundo rustica* : A Meta‐Analysis.” Biological Reviews 92, no. 3: 1582–1600. 10.1111/brv.12297. 27615554

[mec70496-bib-0069] Romano, A. , M. Romano , M. Caprioli , et al. 2015. “Sex Allocation According to Multiple Sexually Dimorphic Traits of Both Parents in the Barn Swallow ( *Hirundo rustica* ).” Journal of Evolutionary Biology 28, no. 6: 1234–1247. 10.1111/jeb.12650. 25913917

[mec70496-bib-0070] Romano, A. , N. Saino , and A. P. Møller . 2017. “Viability and Expression of Sexual Ornaments in the Barn Swallow *Hirundo rustica* : A Meta‐Analysis.” Journal of Evolutionary Biology 30, no. 10: 1929–1935. 10.1111/jeb.13151. 28787531

[mec70496-bib-0071] Saino, N. , S. Calza , and A. Pape Møller . 1997. “Immunocompetence of Nestling Barn Swallows in Relation to Brood Size and Parental Effort.” Journal of Animal Ecology 66, no. 6: 827. 10.2307/5998.

[mec70496-bib-0072] Saino, N. , L. Canova , A. Costanzo , D. Rubolini , A. Roulin , and A. P. Møller . 2013. “Immune and Stress Responses Covary With Melanin‐Based Coloration in the Barn Swallow.” Evolutionary Biology 40, no. 4: 521–531. 10.1007/s11692-013-9228-5.

[mec70496-bib-0073] Saino, N. , M. Romano , R. Ambrosini , et al. 2012. “Longevity and Lifetime Reproductive Success of Barn Swallow Offspring Are Predicted by Their Hatching Date and Phenotypic Quality.” Journal of Animal Ecology 81, no. 5: 1004–1012. 10.1111/j.1365-2656.2012.01989.x. 22531043

[mec70496-bib-0074] Saino, N. , M. Romano , M. Caprioli , et al. 2013. “Molt, Feather Growth Rate and Body Condition of Male and Female Barn Swallows.” Journal of Ornithology 154, no. 2: 537–547. 10.1007/s10336-012-0924-1.

[mec70496-bib-0075] Saino, N. , M. Romano , D. Rubolini , et al. 2013. “Sexual Dimorphism in Melanin Pigmentation, Feather Coloration and Its Heritability in the Barn Swallow ( *Hirundo rustica* ).” PLoS One 8, no. 2: e58024. 10.1371/journal.pone.0058024. 23469134 PMC3585210

[mec70496-bib-0076] Saino, N. , D. Rubolini , R. Ambrosini , et al. 2017. “Sex‐ and Age‐Dependent Morphology and Selection on Wing Shape in the Barn Swallow *Hirundo rustica* .” Journal of Avian Biology 48, no. 11: 1441–1450. 10.1111/jav.01469.

[mec70496-bib-0077] Saino, N. , T. Szép , M. Romano , D. Rubolini , F. Spina , and A. P. Møller . 2004. “Ecological Conditions During Winter Predict Arrival Date at the Breeding Quarters in a Trans‐Saharan Migratory Bird.” Ecology Letters 7, no. 1: 21–25. 10.1046/j.1461-0248.2003.00553.x.

[mec70496-bib-0078] Salomons, H. M. , G. A. Mulder , L. Van De Zande , M. F. Haussmann , M. H. K. Linskens , and S. Verhulst . 2009. “Telomere Shortening and Survival in Free‐Living Corvids.” Proceedings of the Royal Society B: Biological Sciences 276, no. 1670: 3157–3165. 10.1098/rspb.2009.0517. PMC2817122 19520803

[mec70496-bib-0097] Schultner, J. , B. Moe , O. Chastel , C. Bech , and A. S. Kitaysky . 2014. “Migration and Stress During Reproduction Govern Telomere Dynamics in a Seabird.” Biology Letters 10, no. 1: 20130889. 10.1098/rsbl.2013.0889. 24429681 PMC3917333

[mec70496-bib-0079] Sheldon, E. L. , J. R. Eastwood , N. Teunissen , et al. 2022. “Telomere Dynamics in the First Year of Life, but Not Later in Life, Predict Lifespan in a Wild Bird.” Molecular Ecology 31, no. 23: 6008–6017. 10.1111/mec.16296. 34850488

[mec70496-bib-0080] Stier, A. , V. A. Viblanc , S. Massemin‐Challet , et al. 2014. “Starting With a Handicap: Phenotypic Differences Between Early‐ and Late‐Born King Penguin Chicks and Their Survival Correlates.” Functional Ecology 28, no. 3: 601–611. 10.1111/1365-2435.12204.

[mec70496-bib-0081] Stoddard, M. C. , and R. O. Prum . 2008. “Evolution of Avian Plumage Color in a Tetrahedral Color Space: A Phylogenetic Analysis of New World Buntings.” American Naturalist 171, no. 6: 755–776. 10.1086/587526. 18419340

[mec70496-bib-0082] Sudyka, J. 2019. “Does Reproduction Shorten Telomeres? Towards Integrating Individual Quality With Life‐History Strategies in Telomere Biology.” BioEssays 41, no. 11: 1900095. 10.1002/bies.201900095. 31577044

[mec70496-bib-0083] Sudyka, J. , A. Arct , S. M. Drobniak , L. Gustafsson , and M. Cichoń . 2019. “Birds With High Lifetime Reproductive Success Experience Increased Telomere Loss.” Biology Letters 15, no. 1: 20180637. 10.1098/rsbl.2018.0637. 30958221 PMC6371901

[mec70496-bib-0084] Taff, C. C. , and C. R. Freeman‐Gallant . 2017. “Sexual Signals Reflect Telomere Dynamics in a Wild Bird.” Ecology and Evolution 7, no. 10: 3436–3442. 10.1002/ece3.2948. 28515879 PMC5433972

[mec70496-bib-0085] Taylor, G. T. , A. McQueen , J. R. Eastwood , et al. 2024. “No Effect of Testosterone or Sexual Ornamentation on Telomere Dynamics: A Case Study and Meta‐Analyses.” Ecology and Evolution 14, no. 3: e11088. 38435019 10.1002/ece3.11088 PMC10905238

[mec70496-bib-0101] Therneau, T. M. 2024. “coxme: Mixed Effects Cox Models.” R Package Version 2.2‐22, https://CRAN.R‐project.org/package=coxme.

[mec70496-bib-0086] Tricola, G. M. , M. J. P. Simons , E. Atema , et al. 2018. “The Rate of Telomere Loss Is Related to Maximum Lifespan in Birds.” Philosophical Transactions of the Royal Society, B: Biological Sciences 373, no. 1741: 20160445. 10.1098/rstb.2016.0445. PMC5784065 29335369

[mec70496-bib-0087] Turner, A. K. 2006. The Barn Swallow. A&C Black.

[mec70496-bib-0088] Vedder, O. , M. Moiron , C. Bichet , et al. 2022. “Telomere Length Is Heritable and Genetically Correlated With Lifespan in a Wild Bird.” Molecular Ecology 31, no. 23: 6297–6307. 10.1111/mec.15807. 33460462

[mec70496-bib-0089] Verhulst, S. , A. Aviv , A. Benetos , G. S. Berenson , and J. D. Kark . 2013. “Do Leukocyte Telomere Length Dynamics Depend on Baseline Telomere Length? An Analysis That Corrects for ‘Regression to the Mean’.” European Journal of Epidemiology 28, no. 11: 859–866. 10.1007/s10654-013-9845-4. 23990212

[mec70496-bib-0090] Vitousek, M. N. , O. Tomášek , T. Albrecht , M. R. Wilkins , and R. J. Safran . 2016. “Signal Traits and Oxidative Stress: A Comparative Study Across Populations With Divergent Signals.” Frontiers in Ecology and Evolution 4: 56. 10.3389/fevo.2016.00056.

[mec70496-bib-0091] Voituron, Y. , O. Guillaume , A. Dumet , S. Zahn , and F. Criscuolo . 2023. “Temperature‐Independent Telomere Lengthening With Age in the Long‐Lived Human Fish ( *Proteus anguinus* ).” Proceedings. Biological sciences 290, no. 1998: 20230503. 37132239 10.1098/rspb.2023.0503 PMC10154926

[mec70496-bib-0092] Webster, M. S. , R. A. Ligon , and G. M. Leighton . 2018. “Social Costs Are an Underappreciated Force for Honest Signalling in Animal Aggregations.” Animal Behaviour 143: 167–176. 10.1016/j.anbehav.2017.12.006.

[mec70496-bib-0093] Wilbourn, R. V. , J. P. Moatt , H. Froy , C. A. Walling , D. H. Nussey , and J. J. Boonekamp . 2018. “The Relationship Between Telomere Length and Mortality Risk in Non‐Model Vertebrate Systems: A Meta‐Analysis.” Philosophical Transactions of the Royal Society, B: Biological Sciences 373, no. 1741: 20160447. 10.1098/rstb.2016.0447. PMC5784067 29335371

[mec70496-bib-0094] Xiong, Y. , J. Melgar , M. Tobler , and D. Hasselquist . 2025. “Assortative Breeding Experiment in a Songbird Suggests Telomere Length Is Determined During Early Life Rather Than at Conception.” Scientific Reports 15, no. 1: 36510. 10.1038/s41598-025-23517-7. 41120532 PMC12540821

[mec70496-bib-0095] Young, A. J. 2018. “The Role of Telomeres in the Mechanisms and Evolution of Life‐History Trade‐Offs and Ageing.” Philosophical Transactions of the Royal Society, B: Biological Sciences 373, no. 1741: 20160452. 10.1098/rstb.2016.0452. PMC5784072 29335379

[mec70496-bib-0096] Zee, R. Y. L. , A. J. Castonguay , N. S. Barton , S. Germer , and M. Martin . 2010. “Mean Leukocyte Telomere Length Shortening and Type 2 Diabetes Mellitus: A Case–Control Study.” Translational Research 155, no. 4: 166–169. 10.1016/j.trsl.2009.09.012. 20303464

